# Cytogenomic characteristics of murine breast cancer cell line JC

**DOI:** 10.1186/s13039-020-00524-z

**Published:** 2021-02-01

**Authors:** Shaymaa Azawi, Martina Rincic, Thomas Liehr

**Affiliations:** 1 Institute of Human Genetics, Jena University Hospital, Friedrich Schiller University, Am Klinikum 1, 07747 Jena, Germany; 2 Croatian Institute for Brain Research, School of Medicine University of Zagreb, Salata 12, 10000 Zagreb, Croatia

**Keywords:** Breast cancer, Murine cell line, JC, Murine multicolor banding (mcb), Array comparative genomic hybridization (aCGH)

## Abstract

**Background:**

Breast cancer (BC), one of the most frequent human tumors, is genetically and histologically heterogeneous. Treatment options can be adapted according to BC subtype. Still, research is necessary to characterize BC biology better and to study potential new treatment options. Murine BC-cell lines can be used as model systems in this respect.

**Results:**

Here for the first time murine BC-cell line JC was cytogenomically characterized as being complex rearranged and near-tetraploid. Multicolor banding and array comparative genomic hybridization were applied and the result was in silico translated to the human genome.

**Conclusions:**

Even though being commercially available, cell line JC was yet not much included in BC-research, most likely due to a lack of cytogenomic data. Thus, here comprehensive data is provided on chromosomal aberrations, genomic imbalances and involved breakpoints of JC cell line. Also JC could be characterized as a model for BC of luminal B type, basal-like tumor rather than for luminal A type.

**Supplementary information:**

The online version of this article (10.1186/s13039-020-00524-z) contains supplementary material, which is available to authorized users.

## Introduction

Breast cancer (BC) is considered to be one of the most aggressive human cancer forms and is leading among cancer-related deaths, especially in females. Survival rates vary across countries depending on diagnostic regimens, awareness of and possibilities to treat this disease [1, 2]. Factors like advanced age, low estrogen-levels, family history of cancer, and certain adverse gene mutations, as well as lifestyle influence incidence of this cancer type [2, 3]. BCs are a heterogenic group of tumors, and thus divided into subtypes, according to their molecular profiles, morphology, and expression of specific biomarkers [4]. Therefore, each subtype has a different prognosis, a specific response to treatment and a defined clinical outcome [4, 5]. All these go together with repeatedly observed aggressive clinical courses and limitations in breast cancer treatment [6]. BC biomarkers include oncogenes and tumor suppressor genes (TCGs), as well as their and related gene-products. Immunohistochemical markers for tumor cell surface or plasma, as well as for growth in this context are estrogen receptor, progesterone receptor, human epidermal growth factor receptor-2 (ERBB2/ HER-2) and epidermal growth factor receptor, cytokeratin 5 and/or nuclear protein Ki67 expression [1, 7]. Based on such expression profiles BC can be classified in (1) luminal A-like, (2) luminal B-like (HER2-positive or HER2-negative), (3) HER2-overexpressing, and (4) triple-negative subtypes [4, 7].

According to subtype, treatment regimens for BC are adapted [7, 8]. After initial surgery, being commonly applied for most BC subtypes chemo- and radio-therapeutic care follow, most of which have severe side effects. Thus, new types of medication being targeted only towards BC cells are still a pressing necessity [9]. The latter can be e.g. approached by including animal, especially murine models, in research to study biological pathways of BC and BC progression [10–12]. Even though murine cell lines are applied in ample research, still most of them are not characterized in detail genetically [1]. This is also valid for the here studied BC cell line JC, which was established in 1978 from a murine BC, developing spontaneously in a 1.5 years old female BALB/c mouse [13]. JC-cells show papillary adenocarcinoma morphology, and cytogenetics done in 1989 revealed only 26–100 chromosomes per cell, and 40 per cell being found in 28 of the 99 evaluated metaphases [14].

Molecular cytogenetics is considered the most practicable technique to characterize genetic alteration in cancer [15]. Thus, here for the first time, the murine BC cell line JC was studied in detail by multicolor-fluorescence in situ hybridization (FISH) using all murine whole chromosome painting (wcp) probes and FISH-based murine multicolor banding (mcb) approach together with array-comparative genomic hybridization (aCGH). The in silico translation performed on the data to determine the corresponding homologous genetic alterations in human BC was done as previously described [16].

## Materials and methods

### Cell lines

The cell line JC was obtained from American Type Culture Collection (ATCCR CRL-2116™, Wesel, Germany). After being adherently grown in RPMI-1640 medium containing 10% fetal calf serum with recommended antibiotics, cells were cytogenetically prepared [17], and in parallel whole genomic DNA was extracted from the same passage of cells [16]. Molecular cytogenetic / FISH analyses (see below) on the cell line-derived chromosomes, and aCGH analyses on the extracted DNA (see below) were done. According to the ethical committee (medical faculty) and the Animal Experimentation Commission of the Friedrich Schiller University, there are no ethical agreements necessary for studies involving murine tumor cell lines like JC.

### Molecular cytogenetics

FISH was performed as previously described [16] using whole chromosome paints (“SkyPaintTM DNA Kit M-10 for Mouse Chromosomes”, Applied Spectral Imaging, Edingen-Neckarhausen, Germany) for multicolor-FISH (mFISH), and murine chromosome-specific multicolor banding (mcb) probe mixes for FISH-banding [18]. At least 30 metaphases were documented and analyzed for each probe set (Zeiss Axioplan microscopy, equipped with ISIS software (MetaSystems, Altlussheim, Germany). Array-based comparative genomic hybridization (aCGH) was done according to standard procedures by “SurePrint G3 Mouse CGH Microarray, 4 × 180 K” (Agilent Technologies) [16].

### Data analysis

Imbalances and breakpoints being observed in JC were determined according to aCGH and mcb data, and aligned to human homologous regions using Ensembl and the UCSC Genome Browser, as previously described [19]. The obtained data were compared to genetic changes known from human BCs according to literature [7, 20–32].

## Results

After wcp-based mFISH analyses, JC presented as relatively stable, hypo-tetraploid cell line with 64-66 chromosomes (result not shown). Overall there were 3 clones, which differed only in few alterations.

Clone 1 was considered as ancestor clone and present in ~17% of the analyzed cells; karyotype: 64-66<4n>,XXX,-X,del(1)(E2),dic(2;9)(A1;A1),-3,-4,del(5)(B),der(6)(:14B→14A1::6A1→6B3::6B3→6B1::6B1→6B3::6B3→6B1::6B1→6B3::16C1→16qter),-7,idic(8)(A1;A1),dic(9;13)(:13A5->13A5:::9A1→9qter),-10,-12,+13,-14,-16,-17,-18,-19.

**Fig. 1:**
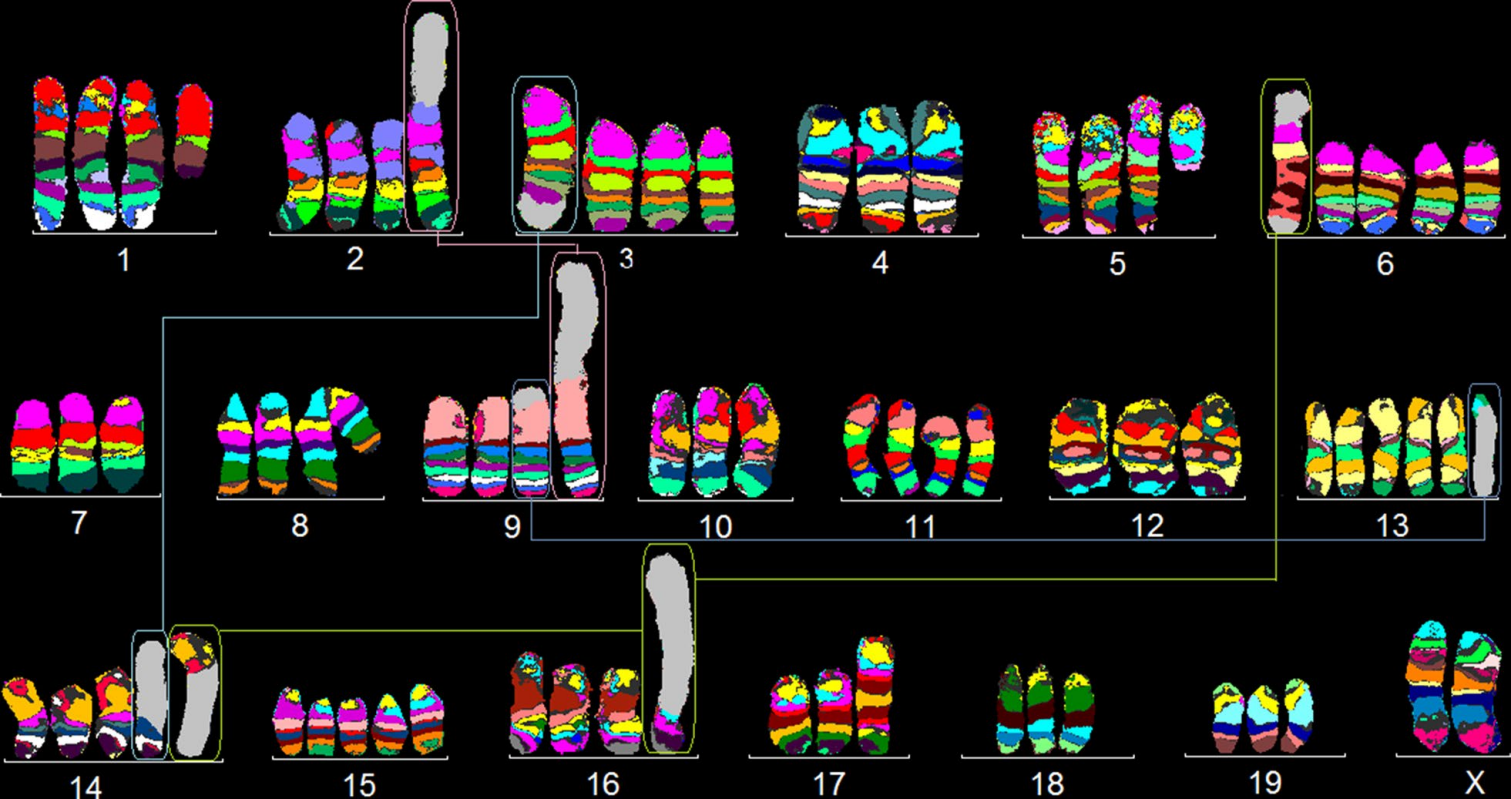
Murine multicolor banding (mcb) was applied on chromosomes of the JC cell line. This figure depicts the summary of 20 chromosome-specific FISH-experiments as typical pseudocolor banding. Derivative chromosomes consisting of different chromosomes are highlighted by frames and shown twice or thrice in this summarizing karyogram

Clone 2 (53.3%) was present in ~53% had the same karyotype shown in Fig. 1 as 64-66<4n>,idem,XX,-X,-X,idem,t(3;14)(H4;D1),+6,+15,dup(17)(A2D1).

Clone 3 (~30% of the cells) had the karyotype:

- 64-66<4n>idem,XX,der(X)(X;?)(pter→A6::?),-X,t(3;14)(H4;D1),+6,der(?)(?;11)(pter→A1::E1→qter),del(13)(A5),dup(17)(A2D1),+15.

**Fig. 2:**
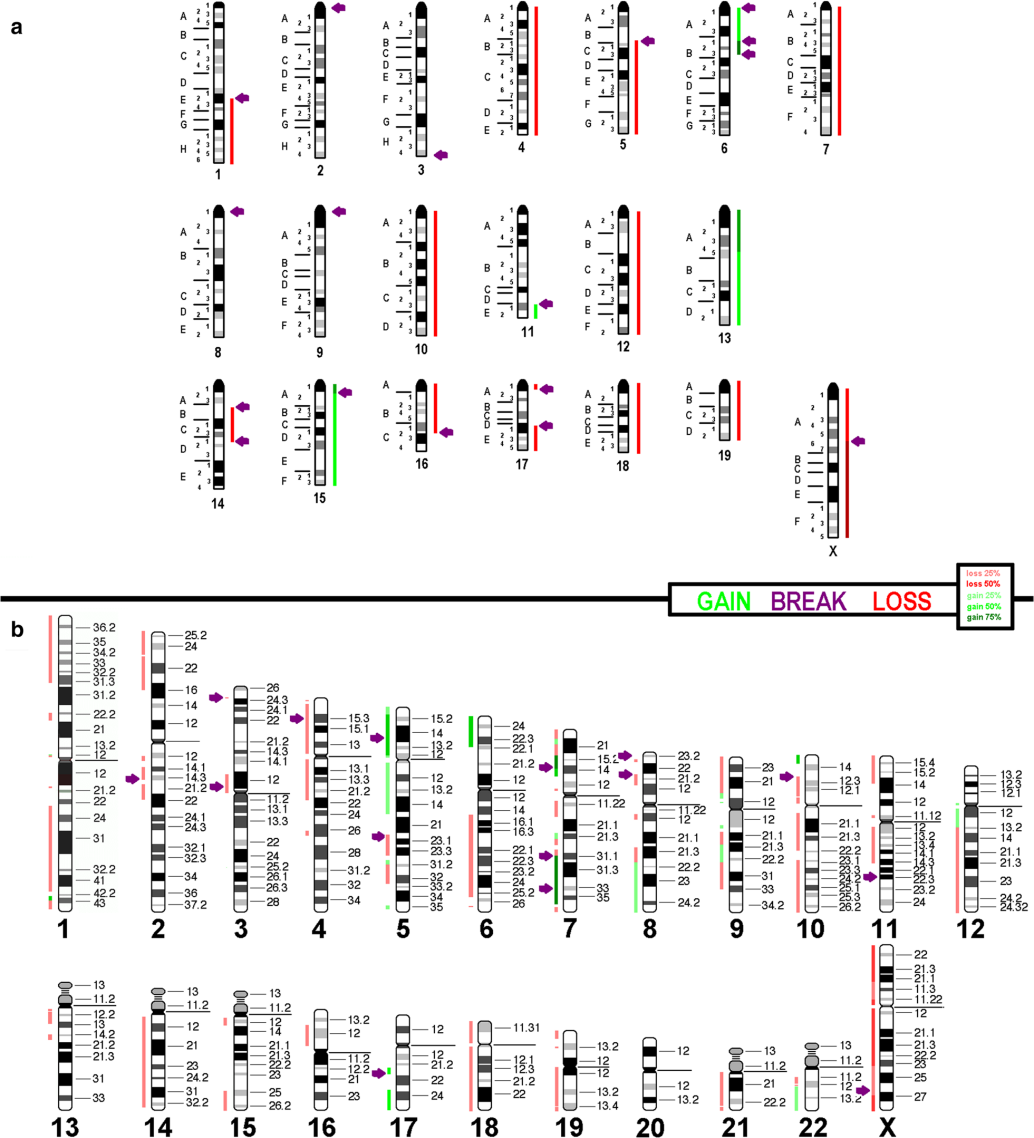
aCGH-results and copy number variations detected in cell line JC are summarized here with respect to a diploid-basic karyotype. Gains are shown as green bars and losses are red, and breaks are registered as arrows. **a** Imbalances found in the cell line depicted along a murine chromosome set. **b** Results translated and projected along the human chromosome set

Overall, FISH-data was in agreement with the aCGH results and copy number alterations and breakpoints are summarized in Fig. 2a. An in-silico-translation of those results to the human genome (only imbalances larger than 3.5 megabase pairs were included) identified the corresponding homologous region in the human genome (Fig. 2b). Genomic details are given in Additional file 1: Table S1.

The corresponding homologous regions for the cell line were compared with common imbalances in human BC, and this revealed copy number variations in regions known to harbor oncogenes and tumor suppressor genes (TSG) with an overlap of 40% (Table 1). The breakpoints of the cell line compared with the chromosomal breaks of human BC overlap to 56% (Table 2). Genetic alterations in cell line JC correlated best with the subtype for human BC acc. to Horlings et al. [32] with basal-like (50%) and luminal B type tumors (67%); no correlation was found with human luminal A type BCs (Table 3).

**Table 1:** Oncogenes and tumor suppressor genes (TSG), related to human BC [20, 21] and their involvement in gains or loss of copy numbers in JC cell line

Oncogenes and TSGs	Gene loci in human	JC
*NRAS*	1p13~22	no CNV
*MSH2*	2p22	loss
*RAF1*	3p25	no CNV
*RARβ2*	3p24	no CNV
*MLH1*	3p21	no CNV
*APC*	5q21	no CNV
*MYB*	6q22-q23	loss
*IGFII*-*R*	6q26	no CNV
*MYC*	8q24	gain
*CDKN2A (p16INK4)*	9p21	no CNV
*PTEN*	10q23	loss
*HRAS*	11p15.5	loss
*FGF3*	11q13	loss
*CCND1*	11q13	loss
*ATM*	11q22	no CNV
*CDKN1B (p27kip1)*	12p13	no CNV
*KRAS2*	12p12.1	no CNV
*BRCA2*	13q12	loss
*Rb1*	13q14	no CNV
*CDH1 (E*-*cadherin)*	16q22	no CNV
*TP53 (p53)*	17p13	no CNV
*BRCA1*	17q21	no CNV
*ERBB2*	17q21	no CNV
*SERPINB5 (maspin)*	18q21	loss
*STK11 (LKB1)*	19p13	loss
SUM of concordance in CNVs of potentially affected regions		**10/25**

*CNV* copy number variant

**Table 2:** Breakpoints in JC compared to the observed acquired breaks in human BCs according to the literature [7, 20–32]: Concordances with human breakpoints are highlighted in bold

Breakpoint acc. to human genome	Human BC	JC
1p33	+	–
1q25.3	+	–
2p23.3	–	**–**
3p12.3	+	–
4p12	–	**–**
4q26	+	–
4q31.23	+	–
4q32.2	–	**–**
5p14.2	+	**+**
5q13.2	+	**+**
5q14.3	+	**+**
6q25.2	–	**–**
7p14.1	–	+
7q31.1	–	**–**
7q36.2	–	**–**
8q23.3	+	**+**
8q24.22	+	**+**
9p24.2	+	–
9p21	+	–
10p11.21	+	–
10q25.1	–	**–**
11p15.5	+	–
12q12.1	+	**+**
12q24.31	+	–
13q21.2	+	–
14q32	+	–
16p12.3	–	**–**
17q21	+	**+**
Xp22.2	–	**–**
Xq23.2	–	**–**
SUM of concordance	**17/30**

**Table 3:** Copy number alterations associated with molecular subtypes of human BC, according to [32], with the copy number variants (CNVs) in cell lines JC

DNA changes		
In BC subtypes	Human BC	JC
**HER2+/ERBB2**		
17q11.1~12	gain	no CNV
17q21.31~23.2	gain	gain but ERBB2 is not included
SUM of concordance		***0/2***
**Basal-like tumors**		
4p15.31	loss	**loss**
5q12.3~13.2	loss	gain
5q33.1	loss	**loss**
6p12.3	gain	no CNV
6p21.1~23	gain	**gain**
8q24.21~24.22	gain	**gain**
10p12.33~14	gain	loss
10q23.33	loss	**loss**
12q13.13	loss	gain
12q13.3	loss	**loss**
15q15.1	loss	no CNV
15q21.1	loss	no CNV
SUM of concordance		***6/12***
**Luminal A**		
1q21.3	gain	no CNV
1q44	gain	loss
16p13.13	gain	no CNV
16p13.12	gain	loss
16q11.2~13	loss	no CNV
16q22.1-24.1	loss	no CNV
SUM of concordance		***0/6***
**Luminal B**		
1p31.3	loss	**loss**
8p21.2	loss	**loss**
17q23.2	gain	loss
SUM of concordance		***2/3***

Concordances with human CNVs are highlighted in bold

*CNV* copy number variant

## Discussion

BC is one of most frequent tumors in females, genetically heterogeneous and with an often very aggressive course. Therefore, still basic research is necessary to understand better its biology and to find more efficient treatment strategies. Accordingly, especially murine tumor cell lines are favorite model systems to perform basic research studies, as well as such towards testing of new medications [8, 33]. Our group characterized already several murine tumor cell lines by the same test strategy as applied here, and thus delivered previously not available but urgently necessary genetic basic data, including chromosome numbers, observable breakpoints and copy number changes [1, 16, 17, 19, 34, 35]. Here a commercially available murine BC-cell line was characterized for the first time, cell line JC, which was yet rarely used in research. It may be speculated that this was in major parts due to the fact that there was no genetic information available, yet. This gap was closed by the present study now.

JC cell line presents a near-tetraploidy karyotype, which is typically observable, especially in human [36], however, only present in about 50% of murine tumor cell lines [1, 16, 17, 19, 34, 35]. Polyploidy promotes malignant transformation of mammary cells and increases cell resistance to drugs [36]; still, it is also a known adaptation of cells to cell culture conditions. In case of JC cell line the only available previous cytogenetic characterization dates back to 1989; at that stage the cell line was extremely chromosomally instable with chromosome numbers reported between 28 and 99. As ~  30% of the cells had modal chromosome numbers of 40 [14] it must be suggested that since that time a stabilization of the karyotype by tetraploidization took place. However, structurally rearranged chromosomes were not duplicated, as observed in other tetraploid BC cell lines [1]. For observed copy number alterations gain of copy numbers was observed for *MYC* and loss of heterozygosity for tumor suppressor genes *MSH2*, *PTEN* and *BRCA2* were determined here.

Overall, murine cell line JC can has here been genetically characterized and it could be shown to be suited as a model for BC of luminal B type, basal-like tumor. It seems to be not suited as luminal A type BC model.

## Supplementary information

**Additional file 1: Table S1** The regions of gain and loss of copy numbers, as well of breakpoints of balanced rearrangements, observed in JC and the corresponding homologue regions in humans are listed as cytoband and position (GRCh37/hg19).

## Data Availability

All relevant data and material is included in this publication.

## Abbreviations

aCGH: array comparative genomic hybridization
BC: breast cancer
CNV: copy number variant
FISH: fluorescence in situ hybridization
ISCN: International System for Human Cytogenomic Nomenclature
mcb: murine multicolor banding
mFISH: multicolor-fluorescence in situ hybridization
wcp: whole chromosome painting
