# Supplementary material for: Cytogenomic characteristics of murine breast cancer cell line JC
Source: Mol Cytogenet. 2021 Feb 1;14:7. doi: 10.1186/s13039-020-00524-z (PMC7852212; doi:10.1186/s13039-020-00524-z)
Supplement: Supplementary file 1 — Additional file 1: Table S1 The regions of gain and loss of copy numbers, as well of breakpoints of balanced rearrangements, observed in JC and the corresponding homologue regions in humans are listed as cytoband and position (GRCh37/hg19). [file 13039_2020_524_MOESM1_ESM.docx]

**Supplementary Table 1**

| **region** | **gain** | **homologue region in human** | |
| --- | --- | --- | --- |
|  |  | cytoband | position (GRCh37/hg19) |
| 6A1-B1 | 1x | 7q21.2-q21.3  7p22.1-p21.3 | 7:92745197-97502117  7:7132996-12536829 |
| 6B1-B3 | 4x | 7q31.1-q36.1  7q36.1  7p15.3-p14.3 | 7:112138919-149583263  7:150032467-150558657  7:23254035-33103246 |
| 11E1-qter | 1x | 17q21.31-q21.32  17q21.32  17q23.2-q24.1  17q24.1-q24.2  17q24.2-q25.3 | 17:43706746-45150591  17:45188646-45518436  17:60483588-62760387  17:62990972-66110690  17:66224207-81175056 |
| 13A1-A5 | 2x | 10p15.3-p15.1  1q42.3-q43  7p14.2-p13  6p22.3-p22.1  6p25.3-p23  6p23-p22.3 | 10:138698-5865622  1:235330060-240084659  7:36524506-43605930  6:20065223-28502803  6:181261-15099150  6:15104709-20060798 |
| 13A5-qter | 1x | 9q22.1-q22.32  5q35.2-q35.3  5q31.1-q31.2  9q21.32-q21.33  9q22.32-q22.33  9p13.1  9q12-q13  9p11.2  8q22.1  5p15.33-p15.31  5q14.3-q15  5q13.2-q14.3  5q11.1-q13.2  1p11.2  5p12 | 9:91031851-97067712  5:173750964-177039611  5:134073478-137090938  9:86231955-90340399  9:97320957-99417669  9:38810965-40707569  9:65585614-65901647  9:43623473-43941731  8:97247028-97373828  5:191425-7935441  5:84566270-96144383  5:70265557-84371909  5:49569996-68922426  1:121149401-121350677  5:43446298-46118514 |
| 15A1-A2 | 2x | 5p15.31-p12 | 5:8927745-42888975 |
| 15A2-qter | 1x | 8q22.1-q24.3  22q12.3-q13.33  12p11.1  12q12-q13.2 | 8:97446632-146158346  22:35962951-51222438  12:33476533-34210697  12:38607141-55072925 |
| **region** | **loss** | **homologue region in human** | |
|  |  | cytoband | position (GRCh37/hg19) |
| 1E2-qter | 1x | 18q21.32-q22.1  2q14.3  2q14.1-q14.3  2q21.2-q22.1  1q32.1-q32.2  1q21.1  1p11.2  1q23.1-q32.1  Yq11.23  1q43-q44  4q26  1q32.2-q42.13 | 18:58351903-65328593  2:122585948-126347698  2:114436107-122578025  2:133138389-138607743  1:206075775-207534964  1:143881371-144095755  1:120754434-120887322  1:158516903-205922697  Y:28358518-28544030  1:240253393-247125743  4:119339188-119512723  1:207575939-227644727 |
| 4A1-qter | 1x | 8q12.1-q12.3  8q21.3-q22.1  8q12.3  6q14.3-q16.2  9p21.2-p13.1  9q22.33-q33.2  9q21.31-q21.32  9q21.32  9p24.1-p21.2  1p32.1-p31.3  1p36.33-p32.2~1 | 8:56650304-62695565  8:87057363-97246782  8:63094926-64018516  6:87793887-100245013  9:27325073-38472099  9:100037894-123488942  9:82993521-85697078  9:85856924-86154717  9:6847129-27220407  1:59120351-67562260  1:894315-59012766 |
| 5B3-qter | 1x | 4p16.3-p11  4q12-q22.1  1p22.2-p22.1  4p16.3  12q24.33  22q11.23-q12.1  12q23.3-q24.11  12q24.11-q24.31  12q24.31-q24.33  7p11.2  7q11.21-q11.22  7q11.23  7q22.1  7p22.3-p22.1  7q21.3-q22.1  13q12.13-q13.2 | 4:4184743-49083612  4:52689038-89000187  1:89950168-93744300  4:493106-1023731  12:132378991-133522542  22:25201765-29156283  12:108325357-110486420  12:110488793-121497537  12:121577100-132336561  7:56019352-56184138  7:66808098-72045725  7:72536306-76149827  7:99552841-102191754  7:169204-6771649  7:97598308-99229367  13:26784894-34260463 |
| 7A1-qter | 1x | 19q13.42-q13.43  19q13.43  19q13.31-q13.33  19q12-q13.31  19q12  19q13.33-q13.41  16p13.11  11p15.1-p14.3  15q11.2  15q11.2-q13.1  15q13.1-q13.3  15q26.3  15q26.1-q26.3  15q25.3-q26.1  15q25.1-q25.3  11p11.12  11q13.4-q14.3  10p11.21  11p15.4-p15.1  16p13.11  16p13.11-p12.3  16p12.3-p12.2  16p12.2-p11.2  16p11.2  16p11.2  10q26.11-q26.3  11p15.5-p15.4  11q13.3-q13.4 | 19:54368915-57485284  19:58523795-59089552  19:45010010-48707700  19:30093064-44860951  19:28589680-30085362  19:48800017-51921957  16:16252815-16388674  11:17403485-25251145  15:22833222-23086601  15:23914751-28586067  15:29107424-32578594  15:99080385-102265870  15:91593058-99078056  15:85829657-91565912  15:80253398-85682414  11:49250334-49827246  11:71627032-89350901  10:37191655-37402201  11:3631069-17360027  16:15260325-15369270  16:16681590-18325190  16:18608156-21351663  16:21572755-28339524  16:28390845-29030948  16:29661006-31520748  10:121224592-135295738  11:192898-3098752  11:68728143-71212974 |
| 10A1-qter | 1x | 6q22.31-q25.2  6q16.3-q22.1  6q22.1-q22.31  2q12.3-q13  10q21.1-q22.1  22q11.22-q11.23  21q22.3  19p13.12  19p13.3  12q23.3  22q12.3  12q13.2-q23.3 | 6:123289910-154997844  6:100545979-116923157  6:117047677-123151435  2:109065537-110402734  10:55366623-74862972  22:23396488-25031504  21:45278940-48084912  19:15052169-15262910  19:281181-4173052  12:104359309-108176937  22:32783299-33472414  12:55351591-104351507 |
| 12A1-qter | 1x | 2p25.1-p23.3  2p25.1  2p25.1  2p25.3-p25.1  7q22.3-q31.1  7p21.3-p21.1  7q31.1  14q12-q22.1  14q23.1-q32.33  7q36.3  7p21.1-p15.3 | 2:10303009-26361943  2:9354723-9994801  2:9996101-10284917  2:140908-9278318  7:105210238-107772185  7:12561752-19748810  7:107772206-112136146  14:25157192-52251174  14:58666612-106375879  7:157225645-158937901  7:19761201-22528893 |
| 14B-D1 | 1x | 3p25.1  10q11.2-q11.23  10q23.1-q23.2  14q22.1-q23.1  14q11.2-q12  14q12  13q12.12  13q12.11  13q14.2  13q12.13  13q12.12  13q14.2-q14.3  8p23.1  8p21.3-p12 | 3:15245114-16307845  10:46488677-51727392  10:82019368-88976316  14:52688635-58629894  14:20211286-24987352  14:25040539-25149959  13:25188452-25511922  13:20207279-23370461  13:49821990-50161404  13:25685086-26668986  13:23853398-24896355  13:50192169-52356487  8:9744629-11737304  8:20206584-29151199 |
| 16A1-C2 | 1x | 3p12.3-p11.1  21q11.2-q22.3  21q11.2  18p11.21  2q21.1 | 3:75865702-90309600  21:15515528-43438088  21:14535253-14714360  18:15016525-15155234  2:132604281-132757591 |
| 17A1 | 1x | 6q25.2-q25.3  6q27 | 6:155053083-160101646  6:167120855-167552070 |
| 17E1-qter | 1x | 18p11.32-p11.22  2p23.2-p16.3  2p16.3-p16.2  18p11.32 | 18:2534401-9972541  2:29033520-51699597  2:51709987-53282184  18:861722-2534400 |
| 18A1-qter | 1x | 10p11.21  10p12.1-p11.22  10p12.1  10p11.21  18p11.32  18q11.1-q12.3  2q14.3  5q22.1-q22.2  5q31.2-q32  5q22.2-q23.3  5q32-q33.1  18p11.22-p11.21  18q21.31-q21.32  18p11.21  18q12.3-q21.31  18q22.1-q23 | 10:35284099-35521818  10:28950711-32678701  10:27747786-28722506  10:35676708-37094546  18:112543-599224  18:18528605-41073893  2:127805408-128786667  5:110280120-112296881  5:137225085-147624774  5:112310736-130339352  5:147647374-150177176  18:10202644-11518916  18:54267924-58201586  18:11649353-13871680  18:41355914-54244819  18:66339761-78010601 |
| 19A1-qter | 1x | 11q12.1-q13.3  9q21.11-q21.31  2q13  9p24.3-p24.1  10q11.23-q21.1  10q23.2-q26.11 | 11:57844834-68709722  9:69086307-82777364  2:114171139-114321953  9:51374-6659223  10:51917603-54540082  10:89234113-121219507 |
| XA1-A6 | 1x | Xp11.23-p11.22  Xp21.1-p11.23  Xq23-q24  Xq24-q27.1 | X:48262014-51358982  X:37364439-47520178  X:115210308-117585111  X:117586665-140073167 |
| XA6-qter | 2x | Xq27.2-q28  Xq23  Xp22.31-p22.2  Xp22.11-p21.1  Xq11.1-q23  Yp11.2  Xp11.22-p11.21  Xp22.2  Xp22.2-p22.11 | X:140429142-154494231  X:114569624-114885545  X:8784578-9687806  X:23850309-37316857  X:62853720-114517895  Y:4132374-5642381  X:52987493-56318562  X:9688235-9917528  X:10415591-23849592 |
| **region** | **breakpoint** | **homologue region in human** | |
|  |  | cytoband | potential tumour-associated genes |
| 1E2 | del | 2q14.3 | 2:124782864-125672864 (*CNTNAP5*) |
| 2A1 | dic | 10p13 | 10:15144583-15210692 (*NMT2*) |
| 3H4 | t | 1p31.3 | 1:68564142-68698803 (*WLS*) |
| 5B3 | del | 4p15.32 | 4:16503164-16900432 (*LDB2*) |
| 6A1 | t | 7q31.1 | 7:114562209-114659256 (*MDFIC*) |
| 6B1 | dup | 7q33 | 7:135611509-135662101 (*MTPN*) |
| 6B3 | t/dup | 7p14.3 | 7:31790793-32338941 (*PDE1C*) |
| 8A1 | idic | 8p23.2 | 8:2792992-4852494 (*CSMD1*) |
| 9A1 | idic | 11q22.3 | 11:106555148-106889250 (*GUCY1A2*) |
| 11E1 | dup | 17q21.32 | 17:45000499-45124520 (none) |
| 14B | t | 3p25.1 | 3:15602211-15643338 (*HACL1*) |
| 14D1 | t | 8p21.1 | 8:28747911-28922281 (*HMBOX1*) |
| 15A2 | dup | 5p14.1 | 5:26880709-27121257 (*CDH9*) |
| 16C2 | t | 3p12.1 | 3:85008132-86123579 (*CADM2*) |
| 17A1 | dup | No homologues |  |
| 17E1 | dup | 5q22.1 | 5:109624934-110074657 (*TMEM232*) |
| XA6 | del | Xq26.3 | X:136648301-136659850 (*ZIC3*) |
